# Initial investigation of ^18^F-NaF PET/CT for identification of vertebral sites amenable to surgical revision after spinal fusion surgery

**DOI:** 10.1007/s00259-012-2196-7

**Published:** 2012-08-16

**Authors:** Andrew Quon, Robert Dodd, Andrei Iagaru, Marcelo Rodrigues de Abreu, Sergio Hennemann, Jose Maria Alves Neto, Clarice Sprinz

**Affiliations:** 1 Department of Radiology/Division of Nuclear Medicine, Stanford University Medical Center, Palo Alto, USA; 2 Department of Neurosurgery, Stanford University Medical Center, Palo Alto, USA; 3 Department of Radiology/Nuclear Medicine, Hospital Mãe de Deus, Porto Alegre, Brazil; 4 Department of Orthopedic Surgery, Hospital Mãe de Deus, Porto Alegre, Brazil; 5 300 Pasteur Drive H-2226, Stanford, CA 94402-5281 USA

**Keywords:** Fluoride PET/CT, Spine, Spinal fusion

## Abstract

**Purpose:**

A pilot study was performed in patients with recurrent back pain after spinal fusion surgery to evaluate the ability of ^18^F-NaF PET/CT imaging to correctly identify those requiring surgical intervention and to locate a site amenable to surgical intervention.

**Methods:**

In this prospective study 22 patients with recurrent back pain after spinal surgery and with equivocal findings on physical examination and CT were enrolled for evaluation with ^18^F-NaF PET/CT. All PET/CT images were prospectively reviewed with the primary objective of identifying or ruling out the presence of lesions amenable to surgical intervention. The PET/CT results were then validated during surgical exploration or clinical follow-up of at least 15 months.

**Results:**

Abnormal ^18^F-NaF foci were found in 16 of the 22 patients, and surgical intervention was recommended. These foci were located at various sites: screws, cages, rods, fixation hardware, and bone grafts. In 6 of the 22 patients no foci requiring surgical intervention were found. Validation of the results by surgery (15 patients) or on clinical follow-up (7 patients) showed that ^18^F-NaF PET/CT correctly predicted the presence of an abnormality requiring surgical intervention in 15 of 16 patients and was falsely positive in 1 of 16.

**Conclusion:**

In this initial investigation, ^18^F-NaF PET/CT imaging showed potential utility for evaluation of recurrent symptoms after spinal fusion surgery by identifying those patients requiring surgical management.

## Introduction

The primary objective of this initial study was to evaluate ^18^F-sodium fluoride (^18^F-NaF) PET/CT scanning for helping to correctly manage patients after spinal fusion surgery (arthrodesis). Spinal fusion is a common procedure performed to treat a variety of spine conditions including degenerative disease, spondylolisthesis, and vertebral deformities [1, 2]. After spinal fusion, suboptimal outcomes may be caused by infection, hardware loosening, non-union of fused vertebrae, and/or incomplete growth of bone grafts.

Patients who have recurrent symptoms caused by these complications after spinal fusion surgery undergo standard evaluation by clinical examination and conventional imaging. However, whether a patient requires surgical intervention is difficult to ascertain because CT and/or MRI will often show extensive and nonspecific postoperative changes [3, 4]. The physiology of ^18^F-NaF is similar to that of ^99m^Tc-MDP used in traditional bone scanning. However, ^18^F-NaF PET/CT is significantly faster (both uptake and acquisition), provides superior spatial resolution, and is widely available in the US and Europe [5–7].

This was a pilot investigation of the ability of ^18^F-NaF PET/CT to identify spinal sites requiring surgical revision after fusion surgery and therefore help orthopedists stratify patients into surgical or conservative management. Validation of the PET/CT results was based on findings on surgery or clinical follow-up of at least 15 months.

## Materials and methods

### Patients

This study was conducted as a clinical trial and was fully compliant with the approval from the ethics committees at our respective institutions. This trial was a single-cohort prospective study of consecutive patients enrolled at two institutions between April 2009 and August 2011. The inclusion criteria consisted of identifying patients who had recurrent symptoms after prior spinal fusion surgery. Additionally, these patients had had a clinical evaluation, physical examination and CT scan that had failed to identify a specific contributing source of the symptoms, and the course of management remained uncertain. Other imaging such as MRI, bone scanning and plain radiography were not required. However, if a patient had undergone these examinations, then the inclusion criteria stipulated that these also did not adequately elucidate the course of management. Pediatric patients, pregnant women and patients unfit for additional surgery were excluded.

Included in the study were 22 patients (16 women, 6 men; age range 36–80 years) with recurrent back pain after spinal surgery and an equivocal physical examination and conventional imaging. The patients underwent ^18^F-NaF PET/CT at Mãe de Deus Hospital in Porto Alegre, Brazil (18 patients) and Stanford University Hospital (4 patients). All ^18^F-NaF PET/CT scanning was performed at least 4 months after the most recent surgery (range 4–96 months after surgery, median 21 months; Table 1) and all scanning was performed within 4 months of presentation with recurrent symptoms.

**Table 1:** Key findings in all patients

Patient	Time since surgery (months)	Imaging results^a^			Clinical follow-up		Pain score^b^		Statistical category^c^
		NaF PET findings	SUVmax	Fusion CT	Findings on surgical exploration	Conservative management	Prior to treatment	At 15-month follow-up	
1	8	Abnormal activity around cage and hardware at L4-L5	13.4	Subchondral sclerosis	Cage failure and screw loosening		4	1	True positive
2	12	Increased activity at bone graft	18.1	Linear radiolucency at graft	Screw loosening and bone graft fracture		4	2	True positive
		Focus at L4 right screw	20.3	Radiolucency around screw					True positive
3	60	Focus at L3-L4 screw	12.1	Radiolucency around screw	L3-L4 screw loosening		4	0	True positive
4	12	Intensely increased activity at left L4 bone graft	18.5	Sclerotic changes in bone graft	Paravertebral bone graft fracture		4	1	True positive
5	36	Normal	6.2 (at cervical screws)	No obvious abnormalities requiring surgery		Regional anesthetic block and clinical surveillance; long-term follow-up without pain	4	0	True negative
6	48	Normal	5.8 (at upper thoracic screws)	No obvious abnormalities requiring surgery		Regional anesthetic block and clinical surveillance; long-term follow-up without pain with no intervention	4	0	True negative
7	26	Abnormal activity at bone graft right L5-S1	22.2	Heterogeneous bone sclerosis	Pseudoarthrosis (abnormal mobility) in bone graft on right		4	2	True positive
8	8	Abnormal activity at bone graft L5	12.8	Likely fracture on the right side of graft	Paravertebral bone graft fracture and loosening of right screw in S1		4	0	True positive
		Asymmetric focus at right S1 screw	11.2	Radiolucency around screw					True positive
9	12	Increased uptake around cage at L5-S1	13.3	Subtle increased sclerosis at cage tip	Pseudoarthrosis in the bone graft; fixation cage hardware at L5 loose		4	0	True positive
		Abnormal activity at left L5 bone graft	14.2	Bone reabsorption in region around graft					True positive
10	11	Increased uptake between right paravertebral graft and right iliac bone	23.5	Abnormal bone resorption	Poor graft healing and necrosis at right iliac bone causing an abnormal neojoint		4	0	True positive
11	36	Mild increased uptake at right L4 screw within normal limits	6.9	Completely normal L4 screw		Regional anesthetic block and clinical surveillance; good clinical response in long term	4	1	True negative
12	17	Mild activity in L2 vertebral body within normal limits	5.9	Completely normal L2 vertebral body		Clinical surveillance; improved symptoms; without pain at time of report	4	2	True negative
13	4	Increased uptake in left bone graft L4-L5	16.3	Heterogeneous bone at L4-L5	Bone graft biopsy revealed normal bone		4	N/A^d^	False positive
14	72	Increased activity in bone graft at L5-S1	21.2	Arthrosis at facet joints L5-S1	Pseudoarthrosis at L5-S1 graft site		4	1	True positive
15	17	Borderline abnormal focus at proximal rod on right side of L3 likely within normal limits	6.2	Mild osteolysis at L3		Regional anesthetic block with good clinical response; symptoms resolved without further intervention	4	0	True negative
16	96	Increased uptake in left L5 screw	7.4	Lucency around L5 screw	Graft fracture and distal screw loosening		4	0	True positive
		Intensely increased activity at L5-S1 bone graft	13.8	Possible graft fracture					True positive
17	13	Focal increased uptake in proximal cage hardware	19.8	Mild linear bone resorption	Posterior approach showed proximal and distal cage loosening		4	1	True positive
18	23	Focal increased uptake in middle of hardware at C4	18.7	Very mild lucency around hardware at C4	Micromobility and cage loosening consistent with psuedarthrosis at C4		4	1	True positive
19	8	Increase uptake in cage at C5-C6	13.4	Very mild lucency zone related to bone graft maturation or cage mobility	Cage loosening		4	0	True positive
20	24	Intense focus right side of cage at L5-S1	20.7	Signs of loosening at cage	Follow-up examination showed worsening pain; surgeon’s impression was hardware loosening because new CT scan showed right bone graft reabsorption and lucency at screw; patient averse to surgery		4	1	True positive
		Increased uptake in S1 screws	11.5	CT with lucency area at S1 screws					True positive
21	19	Normal	7.3 at L4-L5 hardware	No obvious abnormalities requiring surgery		Clinical surveillance; follow-up clinical evaluation showed low back pain related to sacroiliitis unrelated to prior surgery	4	2	True negative
22	72	Intense focus at right L4 screw/bone graft	21.1	Subtle cortical resorption around screw/graft area	Vertebral body necrosis at L4-L5 with screw loosening		4	0	True positive

^a^In patients with more than one abnormal focus each lesion is listed separately

^b^Graded: 0 no pain, 1 mild pain, 2 moderate pain, 3 severe pain, 4 severe and debilitating pain

^c^Statistical category assigned to each lesion. If a patient had at least one true-positive finding on surgery, then the imaging results were considered true positive in the patient-by-patient analysis

^d^Developed bladder cancer

### Scanning

The PET/CT system used at Mãe de Deus Hospital was a Phillips Gemini TF scanner with a Brilliance 16-slice CT scanner and at Stanford University Hospital was a GE Discovery 600 PET/CT system with a 16-slice CT scanner. The CT part of the PET/CT acquisition was designed to be equivalent to a stand-alone diagnostic CT protocol optimized for bone/spine imaging: 1.25–2 mm slice thickness, 1 mm increment, 100–140 kV, 180–230 mAs, and matrix size 512 × 512 pixels. Intravenous contrast agent was not utilized. The PET scanning protocol included injection of 222–370 MBq (6–10 mCi) ^18^F-NaF followed by 45 min for tracer uptake. PET scanning consisted of 150–250 s per bed position acquisition time. The number of bed positions varied from five to eight depending on the amount of spine to be covered. Images were reconstructed using the ordered subset expected maximization algorithm with four to eight iterations.

### Interpretation

A nuclear medicine physician and a radiologist with musculoskeletal expertise reviewed all PET/CT images together with the primary objective of identifying or ruling out the presence of lesions amenable to surgical intervention. The most important feature for identifying an abnormality requiring surgery was focal and well-circumscribed activity clearly above the background spine that coregistered on the CT image with sites typical for hardware failure or pseudoarthrosis (such as the ends of fixation rods and cages, screw shafts, bone grafts). Foci without any significant CT abnormality were downgraded in suspicion. Standardized uptake values (SUV) were used to compare individual foci against background spine activity, but an absolute threshold level for positivity was not used. Clinical history and prior CT imaging were also taken into consideration. PET images not corrected for attenuation were analyzed when needed to identify attenuation artifacts caused by the metallic implants. Incidental foci such as osteophytes, benign bone lesions, and mild degenerative changes were noted but were not formally tabulated. Results of ^18^F-NaF PET/CT scanning were then communicated to the referring orthopedist and tabulated in detail for analysis and validation (Table 1).

### Clinical management and outcome measurement

Based primarily on ^18^F-NaF PET/CT imaging, the patient then underwent either surgical exploration (with possible intervention), or conservative management. Surgical exploration consisted of the orthopedic surgeon probing and manually testing the exact region for loosening and hardware failure at sites of abnormal tracer activity. Further, extracted hardware and bone tissue underwent histopathological analysis for evidence of infection or bone necrosis. Conservative management included: regional anesthetic nerve blockade (to provide palliative short-term relief), physical therapy, medication, and bed rest.

PET/CT abnormalities were considered true positive if they were confirmed on surgery or if by the 15-month clinical follow-up other adjunctive examinations and data were wholly consistent with a surgically relevant lesion. PET/CT foci were considered false positive if no operable abnormality was found on surgery or if symptoms improved without surgical intervention. Negative PET/CT scans were considered true negative if symptoms improved with nonsurgical management and/or were corroborated as stable or resolved on adjunctive imaging examinations such as CT and MRI. Negative PET/CT scans were considered false negative if surgical intervention was ultimately required.

The intensity of the patient’s pain was graded from 0 to 4 prior to initiation of treatment and at the 15-month follow-up as follows: 0 no pain, 1 mild pain, 2 moderate pain, 3 severe pain, 4 severe and debilitating pain.

### Statistics

A standard 2 × 2 contingency table was used to calculate the sensitivity and specificity on a patient-by-patient basis. Findings on surgery or the 15-month clinical follow-up were utilized as the gold standard.

## Results

**Fig. 1:**
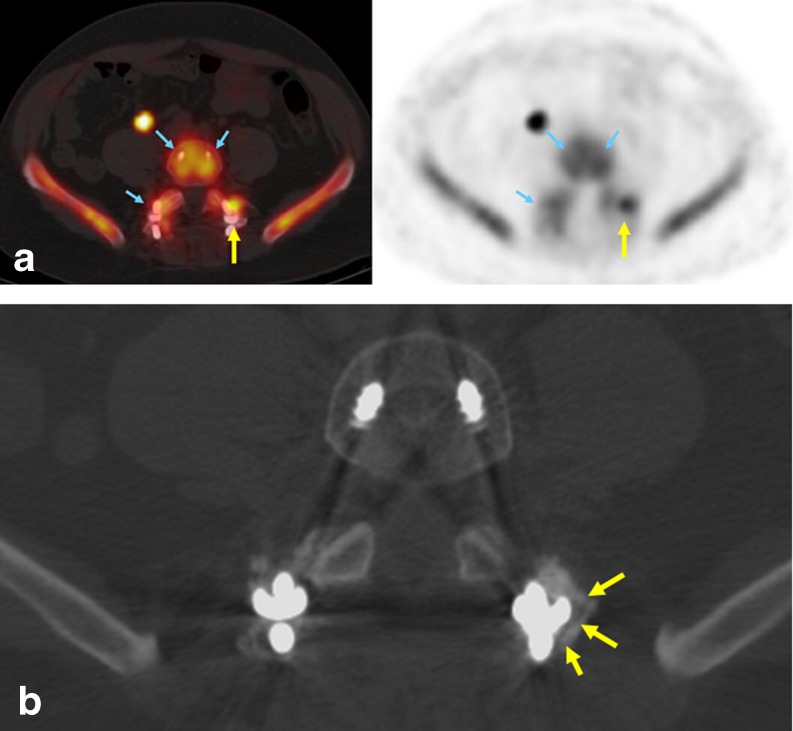
PET/CT images in patient 16 **a** Intense and asymmetric ^18^F-NaF activity is apparent in the left L5 screw (*yellow arrow*, SUV 7.4). Relatively normal tracer activity is apparent in the other hardware (*blue arrows*, SUV 5.2–5.5). Left L5 screw loosening was confirmed on subsequent surgical exploration. **b** Zoomed CT image of the left L5 screw demonstrates possible radiolucency around the screw (*yellow arrows*)

**Fig. 2:**
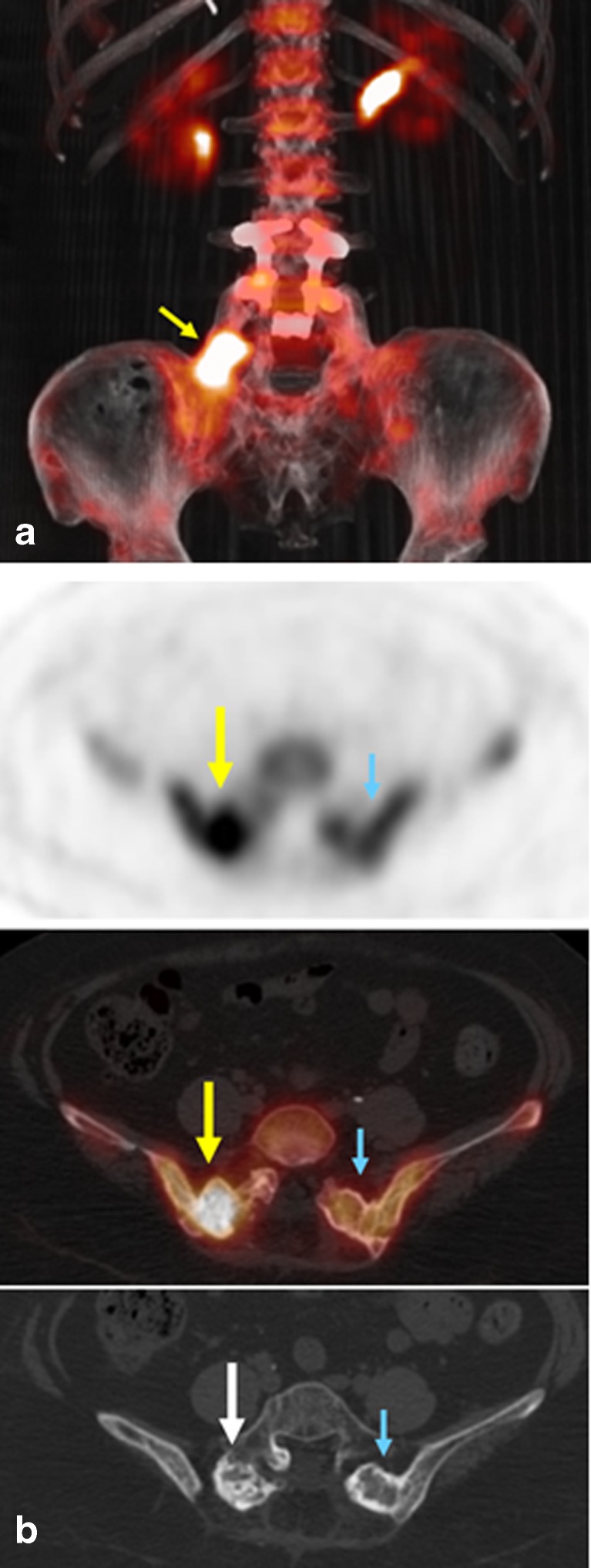
PET/CT images in patient 7. **a** Anterior view 3-D fusion PET/CT maximal intensity projection image demonstrates intensely abnormal activity at the right side of L5-S1 (*yellow arrow*) that was confirmed to be bone graft instability requiring surgical revision. **b** PET, PET/CT fusion, and CT images show markedly asymmetric activity (*yellow arrows*) at the right L5-S1 lamina and facet which is the site of a prior bone graft. Relatively normal activity is apparent within the bone graft located on the contralateral side (*blue arrows*). Increased sclerosis is apparent on the CT image (*white arrow*) at the right L5-S1 graft compared to the left that was initially described as postoperative changes (and difficult to differentiate from graft failure) on the initial CT scan

In total, 116 patients were evaluated for recurrent pain during the study period. Of these, standard clinical evaluation and imaging produced an adequate management plan in 64 patients (55 %) while 52 patients (45 %) did not have conclusive results and met the inclusion criteria for ^18^F-NaF PET/CT. Overall, 22 patients were enrolled and consented to the study while the remaining 30 did not consent to imaging for a variety of reasons including fear of extra radiation, schedule conflict, and/or additional time commitment. Some patients had very long-lasting relief from their original surgery while others presented soon after surgery with new or recurrent symptoms. Also not unexpectedly, the symptomatology may have differed at the initial spinal fusion and at the time of the follow-up PET/CT examination including the exact location and/or the descriptive quality of the complaint. Otherwise, the patient characteristics did not change. All patients had prior multilevel hardware placement exhibiting varying degrees of abnormal anatomical findings where postsurgical healing versus pathological findings could not be clearly differentiated on CT alone. The interpreting physicians found that 16 of the 22 patients had an ^18^F-NaF PET/CT scan that had at least one abnormality amenable to surgical intervention (Figs. 1 and 2). A total of 21 foci were identified amongst these 16 patients. The abnormal foci were located at the following sites: screws (six), cages/rods/fixation hardware (six), and bone grafts (nine) (Table 1). The maximum SUV (SUVmax) of these foci ranged from 7.4 to 23.5 (SUVmax of all foci: average 15.9, median 14.2). The background SUVmax was recorded from the closest comparable vertebral structure without a lesion and ranged from 4.4 to 5.9 (average 5.2, median 5.4).

Of the 16 patients with abnormal PET/CT results, 15 went on to surgical exploration. The orthopedist found abnormalities in 14 of 15 of these patients that correlated precisely with the PET/CT findings and led to repair of the hardware or spine. In one patient (patient 13, Table 1) a biopsy of a bone graft revealed normal bone growth and the PET/CT result was therefore false positive (Table 1). One patient (patient 20, Table 1) with PET/CT abnormalities was not receptive to surgical intervention and was therefore followed clinically rather than subjected to surgery (Table 1). A repeat CT scan at 15 months revealed a graft fracture and hardware loosening and the PET/CT result was therefore true positive.

Of the 22 patients, 6 did not have any foci on ^18^F-NaF PET/CT that appeared amenable to surgery and conservative treatment was recommended. In these normal-appearing patients, the SUVmax was recorded from skeletal sites that had undergone surgery, and the values ranged from 5.8 to 7.3 (SUVmax: average 6.4, median 6.2). The background SUVmax values obtained from comparable spinal structures external to the surgical sites ranged from 5.2 to 6.6 (average 5.6, median 5.4). In all six patients, pain had improved without surgery at the 15-month follow-up and the imaging results were scored as true negative (Table 1).

On a patient-by-patient basis, the sensitivity and specificity of the ability of ^18^F-NaF PET/CT to identify the presence of an abnormality requiring surgical intervention were 100 % (95 % CI 78.2–100 %) and 85.7 % (95 % CI 42.1–99.6 %), respectively. On a lesion-by-lesion basis, 20 of the 21 abnormal foci (in 16 subjects) identified by ^18^F-NaF PET/CT were confirmed as true positive while 1 of 20 was false positive.

## Discussion

^18^F-NaF PET/CT imaging appeared to provide accurate adjunctive information to the standard work-up. Specifically, 14 of 15 patients who underwent surgical exploration based on PET/CT results were confirmed to have lesions requiring surgical intervention by either histopathology or direct manual probing. Overall, 15 of 16 patients were correctly identified by PET/CT as having a lesion requiring surgery after initial evaluation was equivocal regarding the need for surgery. Conversely, all of the six patients with PET/CT results indicating that surgery was not required were found after 15 months of follow-up to have improved (by clinical assessment and reported pain).

**Fig. 3:**
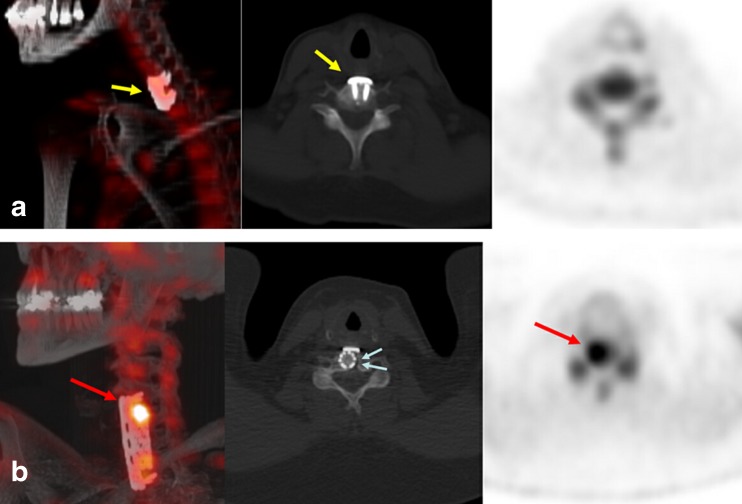
Comparison of patient 5 and patient 17. **a** Patient 5: oblique 3-D PET/CT fusion maximal intensity projection image, axial noncontrast CT image, and axial coregistered PET image show metallic spinal fixation hardware in the cervical neck (*yellow arrows*). There is no evidence of abnormalities on the PET image or the CT image. The patient was therefore referred for conservative management and had eventual improvement of symptoms. **b** Patient 17: oblique 3-D PET/CT fusion maximal intensity projection image, axial noncontrast CT, and axial coregistered PET image show metallic hardware in the cervical neck. An abnormally intense focus is apparent in the proximal spinal fixation cage (*red arrows*), and very subtle radiolucency around the metallic fixation cage (*blue arrows*) is apparent on the CT image. The patient was referred for surgical exploration and hardware loosening and abnormal spinal mobility was confirmed in this region

It should be noted that the timing of postoperative inflammation and its effect on ^18^F-NaF PET/CT cannot be deduced from this project. The single false-positive result in this study occurred in a patient (patient 13, Table 1) who underwent scanning just 4 months after spinal fusion. Nevertheless, most of the postoperative spine in all patients showed normal ^18^F-NaF activity compared to background vertebrae despite extensive hardware placement, suggesting that when given enough time, the tracer has relatively little nonspecific uptake at sites of prior surgery (Figs. 1, 2 and 3).

Our results substantiate and expand on those of a recent study by Fischer et al. that showed the potential of ^18^F-NaF PET/CT for characterizing orthopedic hardware incorporation [8]. However, this prior study lacked outcome data and abnormalities found on ^18^F-NaF PET/CT were described without follow-up information to validate the imaging results. The literature on the use of ^18^F-NaF PET/CT for evaluating the postoperative spine is otherwise very scant. The use of SPECT tracers such as ^99m^Tc-MDP has been described more extensively, although very few studies included integrated SPECT/CT which would be more comparable to PET/CT. A contemporary study by Damgaard et al. using ^99m^Tc-MDP SPECT/CT suggested possible utility for detecting lack of fusion of metallic implants, but this study suffered from the smallness of the cohort which comprised only nine subjects and a retrospective design [9]. A review by Scharf in 2010 also noted the potential for integrated SPECT/CT in a variety of conditions including evaluation of spinal fusion [10]. However, this was not a formal scientific evaluation of data but merely a descriptive report of two case studies. Studies prior to 2000 investigated the utility of ^99m^Tc-MDP SPECT (without integrated CT) more comprehensively [11, 12]. A 1987 study by Slizofski et al. prospectively evaluated 15 symptomatic patients following lumbar fusion and found SPECT to have a sensitivity and specificity of 78 % and 83 %, respectively, for identifying offending osseous sites of recurrent pain [11]. A larger study by Gates and McDonald showed similar results in 63 patients following lumbar surgery [12]. While a statistical analysis was not presented, the authors concluded that SPECT imaging is particularly useful for excluding bony causes of recurrent back pain while positive lesions are helpful for identifying causative abnormalities such as facet arthropathy and pseudoarthrosis. In both studies (Slizofski et al. [11] and Gates and McDonald [12]), validation of SPECT results utilized findings at surgery as well as other imaging. However, the added value of SPECT versus existing conventional imaging, such as CT, plain radiography or MRI, was not directly addressed, and it is not clear whether SPECT would aid treatment decisions beyond conventional work-up. There are no studies that have directly compared ^18^F-NaF PET/CT with ^99m^Tc-MDP SPECT or SPECT/CT in the same cohort of patients although, as stated in the Introduction, ^18^F-NaF PET/CT is superior to ^99m^Tc-MDP SPECT/CT in terms of speed and image quality.

The pilot cohort investigated in our project was small and our high reported sensitivity and specificity should be viewed with caution. Nevertheless, the data and image quality appear promising and a larger clinical evaluation is warranted, including possibly comparing the outcomes of two management approaches in different cohorts, CT and one/CT and one without.

## Conclusion

In this prospective pilot investigation of 22 patients, ^18^F-NaF PET/CT imaging demonstrated potential for aiding management of patients with recurrent pain after spinal fusion surgery by helping to correctly identify those requiring surgical intervention.
